# From pharmacogenetics to pharmaco-omics: Milestones and future directions

**DOI:** 10.1016/j.xhgg.2022.100100

**Published:** 2022-03-16

**Authors:** Chiara Auwerx, Marie C. Sadler, Alexandre Reymond, Zoltán Kutalik

**Affiliations:** 1 Center for Integrative Genomics, University of Lausanne, Lausanne, Switzerland; 2 Department of Computational Biology, University of Lausanne, Lausanne, Switzerland; 3 Swiss Institute of Bioinformatics, Lausanne, Switzerland; 4 University Center for Primary Care and Public Health, Lausanne, Switzerland

**Keywords:** biobanks, bioinformatics, causal inference, electronic health records, genome-wide association studies, multi-omics, pharmacogenetics, pharmacogenome, pharmacogenomics

## Abstract

The origins of pharmacogenetics date back to the 1950s, when it was established that inter-individual differences in drug response are partially determined by genetic factors. Since then, pharmacogenetics has grown into its own field, motivated by the translation of identified gene-drug interactions into therapeutic applications. Despite numerous challenges ahead, our understanding of the human pharmacogenetic landscape has greatly improved thanks to the integration of tools originating from disciplines as diverse as biochemistry, molecular biology, statistics, and computer sciences. In this review, we discuss past, present, and future developments of pharmacogenetics methodology, focusing on three milestones: how early research established the genetic basis of drug responses, how technological progress made it possible to assess the full extent of pharmacological variants, and how multi-dimensional omics datasets can improve the identification, functional validation, and mechanistic understanding of the interplay between genes and drugs. We outline novel strategies to repurpose and integrate molecular and clinical data originating from biobanks to gain insights analogous to those obtained from randomized controlled trials. Emphasizing the importance of increased diversity, we envision future directions for the field that should pave the way to the clinical implementation of pharmacogenetics.

## Introduction

Through the study of genetic determinants of drug responses, pharmacogenetics (PGx) plays a crucial role in achieving the promises of personalized medicine: providing a medical treatment tailored to one’s genetic background. PGx variants modify a drug’s pharmacokinetics by impacting its absorption, distribution, metabolism, and excretion (ADME), its pharmacodynamics by perturbing proteins involved in the drug’s mechanism of action, or immune regulation.^1^^,^^2^ Understanding these interactions improves treatment outcome by optimizing drug dosage and efficacy while minimizing adverse drug reaction (ADR) risk.

To date, the Clinical Pharmacogenetics Implementation Consortium (CPIC)^3^ reports almost 450 gene-drug interactions. Eighty-three of these, involving 22 genes and 63 drugs, are annotated with the highest level of confidence and have prescription guidelines. These 63 drugs represent a small fraction of approved drugs but form a large portion of prescribed drugs,^4^^,^^5^ so that 35%–65% of the population has been exposed to at least one prescription drug with PGx indication.^6^, ^7^, ^8^ Notwithstanding, clinical implementation of PGx has been slow, with concerns regarding clinical validity and cost-effectiveness, infrastructure and data management, lack of awareness and education of health professionals, and ethical and regulatory issues being identified as the main barriers.^9^ To address these concerns, numerous PGx initiatives have been undertaken. So far, these revealed that pre-emptive genetic testing is cost effective in most situations, virtually benefits all given that >95% of individuals present at least one clinically actionable PGx variant, and could improve drug dosage while reducing ADRs.^8^

**Figure 1:**
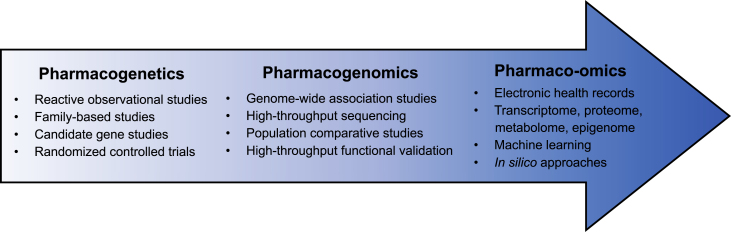
Overview of the chronological development of pharmacogenetics methodology Each section of this review deals with a major PGx milestone: pharmacogenetics, pharmacogenomics, and pharmaco-omics. Listed are some of the main tools and approaches that were instrumental in the development of PGx at various stages. They will be discussed in their respective sections.

As encouraging results from these initiatives bring us a step closer to a widespread clinical implementation of PGx, this review takes a step back to discuss key milestones in the development of PGx methodology aiming at the identification, functional validation, and mechanistic understanding of clinically actionable germline variants (Figure 1). Purposefully omitting advances in oncology, where the consideration of somatic mutations adds an additional layer of complexity, we selected key studies that illustrate these milestones, highlighting how they have shaped PGx. The scientific community increasingly benefits from data streaming from high-throughput experiments, as well as biobanks coupling genetic information to intermediate molecular phenotypes and electronic health records. We emphasize remaining limitations and areas of active research, discussing strategies and methods that have not yet been fully developed and applied to PGx, envisioning the future of the field.

## The origins of modern pharmacogenetics

### The genetic basis of drug responses

The term “pharmacogenetics” was coined in 1959 by Friedrich Vogel^10^ to describe the emerging concept that drug reactions are under genetic control.^11^^,^^12^ This idea originated from a series of observations indicating that individuals react differently when exposed to comparable amounts of a drug. For instance, ∼10% of African Americans develop hemolytic anemia following treatment with the antimalarial drug primaquine, an ADR rarely observed in individuals of European ancestry (MIM: 300908, 305900).^13^ This observation was in line with a genetic origin of the ADR, as narrated by one of the founders of PGx, Arno Motulsky: “since a given gene may be more frequent in certain ethnic groups, any drug reaction that is more frequently observed in a given racial group, when other environmental variables are equal, will usually have a genetic basis.”^12^ The intuition was confirmed decades later, as studies revealed a higher prevalence of causative glucose-6-phosphate dehydrogenase deficiency in Africans,^14^ tightly linking PGx with population genetics from the onset (Box 2).

Box 1 **Glossary**

**Allele:** one of two or more versions of a genetic region.

**Biomarker:** measure of a biological state that is indicative of a physiological or pathological process.

**Diplotype:** combination of two haplotypes present in an individual.

**Epistasis:** phenomenon whereby the phenotypic consequence of a genetic variant is dependent on the presence of one or multiple other genetic variants (non-additive effects; gene-by-gene interactions).

**Expression quantitative trait locus (eQTL):** genetic locus harboring markers that explain a certain proportion of the variance in expression of a given gene.

**Fine-mapping:** statistical approach aiming at determining the most likely causal genetic variant(s) for an observed association signal.

**Forward genetics:** identification of the genetic basis of a phenotype.

**Genetic architecture**: underlying genetic basis of a phenotype (e.g., monogenic, polygenic, ...).

**Genetic reference population:** collection of strains from a model organism that were generated through specific breeding and for which broad genotype and phenotype data are available.

**Genome-wide significance**: threshold of significance that accounts for the fact that genome-wide association studies (GWASs) perform a large number of associations on not fully independent genetic variants. Typically, p ≤ 5 × 10^−8^ is used.

**Haplotype:** group of alleles that are inherited together from a single parent.

**Heritability:** proportion of phenotypic variation in a given population that is due to genetic variation.

**Imputation:** inference of genetic variants that were not directly genotyped using haplotype reference panels. Imputation is possible as blocks of variants in linkage disequilibrium (LD) tend to be co-inherited, so that the presence of a given variant is predictive of the genotype at an adjacent location.

**Linkage disequilibrium (LD):** non-random association of alleles at two or more loci in a population.

**Missense variant:** SNV leading to the substitution of a different amino acid in the resulting protein.

**Monogenic trait:** trait influenced by genetic variants in or near a single gene.

**Oligogenic trait:** trait influenced by genetic variants in or near a few genes.

**Pleiotropy:** phenomenon whereby a single gene or variant affects multiple phenotypes.

**Polygenic score (PGS):** estimate of an individual’s genetic liability to a trait calculated as the weighted sum of phenotype-associated variants, weights being the effect sizes from GWAS summary statistics.

**Polygenic trait:** trait influenced by genetic variants in or near a large number of genes.

**Polypharmacy:** co-prescription of several drugs.

**Population differentiation:** accumulation of differences in allele frequencies between populations with restricted gene flow due to natural selection or genetic drift.

**Population stratification**: presence of sub-populations with different allele frequencies within a seemingly homogeneous population. If not accounted for, population stratification can lead to spurious genotype-phenotype associations.

**Reverse genetics:** identification of the phenotypic consequence(s) of a particular genetic variant.

**Sequencing coverage:** number of unique reads that include a particular base pair.

**Single nucleotide variant (SNV):** substitution of a single nucleotide at a specific genomic location.

**Star allele:** allele following the standardized nomenclature for genetic polymorphisms in pharmacogenetics (PGx) genes. Star alleles are not restricted to single variants but can refer to functional haplotypes of PGx genes.

**Summary statistics:** aggregated GWAS results lacking individual genotype and phenotype data and typically including the variant’s identifier, genomic location, reference and alternative allele, and their respective frequencies, a metrics for the strength of association with incertitude and a p value.

Box 2 **Pharmacogenetics for all**

***Why is diversity relevant?***

Ability to assess the phenotypic impact of a variant depends on its frequency in the studied population. Early on, it was observed that different populations do not respond equally to certain drugs.^13^ Later, microarray studies revealed population differentiation across ADME genes^15^^,^^16^ and sequencing studies generated global maps of PGx allele frequencies.^17^^,^^18^ Following, a gene-based population differentiation method that optimally accounts for rare variants showed that 10% of investigated PGx genes exhibit high population differentiation.^19^

***What are the consequences of lack of diversity?***

Despite growing awareness around the importance of inclusiveness, the majority of genetic studies remain Eurocentric, undermining the ability to discover new PGx variants and implement equitable clinical guidelines. A 2021 systematic review showed that 88% of discovery PGx genome-wide association studies (GWASs) participants were of European ancestry.^20^ This bias is apparent in the largest PGx study to date: 17% of the UK Biobank participants carry at least one undocumented deleterious variant in the 14 analyzed PGx genes, the majority of which being of non-European ancestry.^21^ Similarly, 72% of the participants enrolled in clinical trials leading to US Food and Drug Administration (FDA) drug approval in 2019 were of white ancestry.^22^ Biased and underpowered, such studies miss or underestimate the strength of robust PGx interactions involving variants predominantly observed in minority populations. This results in poor generalizability of findings, which translate into healthcare disparities.^23^ For example, polygenic scores (PGSs) (Polygenic pharmacogenomics) ought to play a major role in personalized medicine but raise ethical concerns due to poor accuracy in individuals of non-European ancestry.^24^

***How can diversity be leveraged to enable new PGx discoveries?***

Studies discussed in this review illustrate how increased diversity can benefit PGx. Including participants of both European and African ancestry, it was shown that the strength of association between hepatitis C treatment efficacy and *IFNL3* genotype was comparable in both populations and that the increased prevalence of the unfavorable *IFNL3* genotype in Africans explained a large fraction of the differences in treatment responsiveness across groups (The first pharmacogenomics GWAS).^25^ In another example, three out of five regions associated with bronchodilators response in asthmatic children were discovered by analyzing minority populations. However, these proved difficult to validate due to the absence of replication cohorts (The human pharmacogenomic landscape).^26^ A third study proposes a mechanistic model to improve warfarin dose prediction by analyzing both European and African American cohorts (Molecular modelling of pharmaco-omics interactions).^27^ Trained on each ancestry group, the model was used to predict maintenance dose in an ancestry-matched and cross-ancestry manner, yielding improved results compared with existing algorithms. This study highlights how a mechanistic approach to modeling combined with a limited number of samples of different ancestries allows to broaden model applicability to other ethnic groups. Overall, inclusiveness can be applied to a wide range of methodologies, leading to knowledge that benefits all.

Acknowledging the role of genetics in drug response promoted usage of classical human genetics tools to the study of PGx.^28^ Twin studies could verify that a drug response was under genetic control by estimating its heritability, while family studies allowed inferring inheritance patterns of drug responses. For instance, inability to metabolize the antihypertensive drug debrisoquine^29^ and the sodium channel blocker sparteine^30^ follows an autosomal recessive pattern of inheritance (MIM: 608902). Inability to metabolize these compounds is strongly correlated and was believed to be controlled by the recently discovered cytochrome P450 (CYP) system.^31^^,^^32^ As oxidative metabolism of other drugs seemed unaffected,^31^ it suggested that only one enzyme of the CYP system was dysfunctional. Overall, these pioneering studies had the merit to confirm the underlying genetic basis of differential drug response, forming the foundations of PGx.

### The candidate gene approach

A drawback of early PGx studies was their inability to map differential drug responses. Progress in molecular biology and improved understanding of cellular and physiological processes set the stage for candidate gene studies (CGSs), which use prior knowledge to identify PGx interactions typically involving common or large-effect-size variants. After selecting candidate genes based on reported roles in drug ADME or relevant biological pathways, association between drug response and genetic variants within these genes is assessed.^33^ For instance, the etiology of the debrisoquine and sparteine non-metabolizer phenotype was elucidated by purifying the relevant CYP^34^ and cloning the relevant alleles.^35^ This gene later came to be known as *CYP2D6* (MIM: 124030), one of the best studied PGx genes. Human *CYP2D6* is part of a family of 57 putatively functional genes, a dozen of which being responsible for the oxidative biotransformation of 70%–80% of all drugs,^36^ arguably making them the most important PGx gene family. Other CGSs elucidated the genetic cause of abacavir hypersensitivity (MIM: 142830), a severe multisystemic response experienced by ∼5% of human immunodeficiency virus (HIV)-infected patients treated with the antiviral drug.^37^ Suspicion of a genetic origin for the ADR led two groups to assess variants in genes involved in abacavir metabolism and immune response, jointly identifying *HLA-B∗5701* as the main genetic risk factor for abacavir hypersensitivity.^38^^,^^39^

These success stories sparked the idea that new PGx knowledge could be brought into the clinic to optimize treatment. Given genotyping costs in the early 2000s,^40^ it was crucial to validate interactions before adopting systematic screening. Most PGx interactions unraveled by CGS resulted from retrospective case-control studies comparing the frequency of suspected genetic variants between a group that experienced the drug response and a group of matched controls that did not.^41^ Despite being cost effective, their retrospective nature makes them prone to various types of biases. They also suffer from general challenges associated with epidemiological study design, such as improper definition of the outcome phenotype, lack of power due to inadequate sample size, or failure to control for stratification or pleiotropy.^41^ Hence, many associations failed to replicate and further proof was warranted prior to clinical implementation.

### Randomized controlled trials

**Figure 2:**
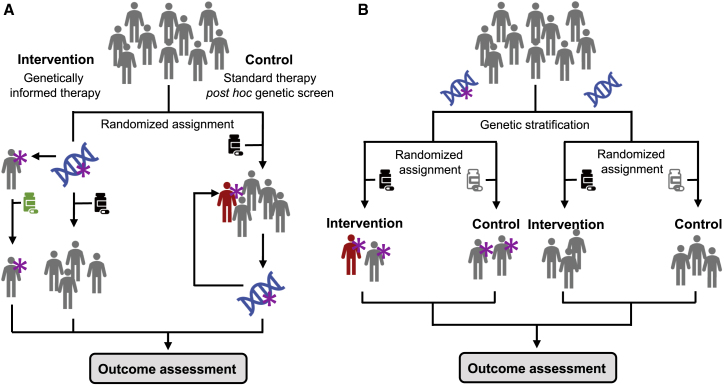
Possible study designs for pharmacogenetic randomized controlled trials (A) Participants are randomly assigned to an intervention or control group. In the former, participants are screened for the presence of the PGx variant of interest (star). Negative individuals receive standard treatment (black), while positive ones receive an adapted alternative treatment (green). In the control group, all individuals receive conventional treatment and genetic screening is performed *post hoc*. The number of participants exhibiting the response of interest (red) is assessed and compared among groups. (B) Participants are stratified based on the presence or absence of the PGx variant of interest. In each strata, participants are randomly assigned to an intervention group, which receives standard treatment (black) and a control group, which receives a placebo or alternative treatment (white). The number of participants exhibiting the response of interest is assessed and compared among groups and strata.

Randomized controlled trials (RCTs) have become the gold standard of evidence-based medicine over the course of the 20^th^ century.^42^ PGx RCTs allow measuring the effects of the genotype, treatment, and their interaction, providing causal evidence for the use of a genotype-informed treatment plan.^41^ Ideally, participants are randomized to receive either genotype-guided or conventional therapy (Figure 2A). Alternatively, when the genotype of interest is rare or when several treatments are to be compared, participants can be stratified based on their genotype and randomized within each stratum (Figure 2B). Following two independent reports of *HLA-B∗5701* as the main genetic risk factor for abacavir hypersensitivity,^38^^,^^39^ a prospective RCT set out to investigate the clinical benefits of *HLA-B∗5701* screening by randomizing ∼2,000 HIV-positive patients according to the design of Figure 2A.^43^ While all participants in the control group received abacavir, individuals in the intervention group were genotyped so that only *HLA-B∗5701*-negative patients received the drug. The study demonstrated that pre-emptive screening reduced the incidence of clinically diagnosed abacavir hypersensitivity from 7.8% to 3.4% (p < 0.001) and eliminated immunologically confirmed abacavir hypersensitivities (2.7% in controls; p < 0.001),^43^ prompting the establishment of clinical screening guidelines.^44^ Recently, the impact of these guidelines was evaluated, showing that, since their introduction, the proportion of patients undergoing pre-emptive screening steadily increased, while ADR incidence decreased 6-fold.^45^

RCTs provide strong support of causality between an exposure and an outcome, but their role in PGx has been called into question.^23^^,^^46^ RCTs present multiple limitations, often related to their high costs. Systematic investigation of 138 clinical trials estimated the median cost of a trial to $19 million,^47^ with costs increasing with participant number and the inclusion of a randomized control group. Studying rare PGx variants or drug response requires large sample sizes and quickly becomes financially unsustainable. This problem is illustrated with the TAILOR-PCI RCT that found no statistically significant reduction in major adverse cardiovascular events (MACEs) (12 months follow-up) upon *CYP2C19*-guided prescription of anti-platelet medication (hazard ratio = 0.66; 95% confidence interval [CI]: 0.43–1.02; p = 0.06).^48^ Clopidogrel is the most widely prescribed anti-platelet medication,^49^ but due to genetically driven variability in drug response, it is suspected to increase the risk for MACEs.^50^ As *CYP2C19* (MIM: 124020, 609535) is required to activate clopidogrel,^51^ *CYP2C19* loss-of-function (LOF) carriers in the genotype-guided group were prescribed ticagrelor instead of clopidogrel.^48^ Despite its large size (n = 5,302), the trial was only powered (85%) to detect a minimum hazard ratio of 0.50, so that the detected trend did not reach significance.^48^ Another limitation is that only two LOF alleles were considered, although recent evidence demonstrated a polygenic architecture of clopidogrel response (Polygenic pharmacogenomics).^52^ Considering these results, the US Food and Drug Administration (FDA) does not require genetic testing prior to clopidogrel treatment initiation, nor do the American College of Cardiology and European Society of Cardiology recommend routine *CYP2C19* testing. Notwithstanding, recent meta-analyses suggest that *CYP2C19*-guided prescription could identify patients benefiting from alternative anti-platelet medication,^53^^,^^54^ prompting the CPIC to update its recommendations in 2022, thereby providing guidance to clinicians on how to interpret *CYP2C19* genetic test results.^55^ Concurrently, a subset of the TAILOR-PCI RCT has increased the follow-up period to 24 months, additionally performing a feasibility study to assess digital solutions allowing more efficient and cost-effective patient follow-up and study design.^56^ Besides the costs and hurdles associated with generating prospective evidence of clinical utility, there is a lack of incentive for the pharmaceutical industry to conduct PGx RCTs, as patents for most drugs with PGx guidelines—including clopidogrel—have expired, making them unprofitable.^2^^,^^23^ Another recurrent issue is the lack of diversity (Box 2). Finally, PGx RCTs are associated with serious ethical concerns: Rarely performed without prior knowledge, some study designs (e.g., Figure 2B) expose patients with a likely actionable variant to a treatment that puts them at risk of ADRs.^23^^,^^41^

Slow clinical implementation of PGx is often credited to lack of supporting evidence by RCTs, which can partially be attributed to the limitations described above. RCTs remain an important epidemiological tool, as demonstrated by the more than 800 PGx trials reported over the last 20 years, of which 155 are still recruiting or ongoing. However, RCTs should not be the sole approach to validate PGx interactions. Proof of efficacy of genetic testing often needs to meet higher standards compared with non-genetic tests, and most physicians unconsciously practice PGx by adjusting patient’s treatments.^46^ To ensure transfer of PGx knowledge from bench to bedside while maintaining rigorous standards of clinical efficacy, it will be important to find more efficient ways to conduct RCTs and broaden the spectrum of accepted proof of evidence. In Leveraging observational data to gain causal insights, we introduce statistical tools leveraging large-scale observational data to approximate the effects of RCT studies.

## The genomic revolution

### The pharmacogenome

Sequencing of the human genome in the early 2000s^57^^,^^58^ brought forward the concept of the druggable genome, originally defined as genes whose product can or are predicted to be bound by small molecules.^59^ Besides providing a blueprint to map genes and variants associated with drug responses, access to the reference genome catalyzed waves of technological progress in genomics (Box 3). A common theme is the possibility to assess more—and potentially novel—variants with higher accuracy and at ever lower prices, leading to the metamorphosis of pharmaco*genetics* into pharmaco*genomics*. Conceptually similar and therefore both referred to as PGx hereafter, pharmacogenomics aims at discovering genetic variants affecting drug response by probing the entire genome, as opposed to a few candidate loci. This progress enabled the exploration of the pharmacogenome, which we define as regions of the human genome that influence response to medication.

Box 3 **Overview of genomic technologies**

**Microarray genotyping:** Genomic variability can be assessed with genotyping microarrays that probe the identity of 250,000–4,000,000 predefined single nucleotide variants (SNVs).^60^ Diverse, high-coverage, haplotype reference panels are used to impute variants at positions that were not genotyped. In spite of increasing the multiple-testing burden, boosting the number of assessed SNVs increases power, improves fine-mapping, and facilitates meta-analyses.^61^ Imputed microarray data allow the assessment of millions of SNVs for a fraction of sequencing costs.

**Short-read sequencing:** A major breakthrough came from the development of high-throughput next-generation sequencing (NGS) methods that implement massively parallel sequencing of short DNA fragments.^62^ Applications include whole-genome sequencing (WGS), which assesses the entire genome, and whole-exome sequencing (WES), which uses capture and enrichment methods to selectively sequence exons, the protein coding regions of the genome. Because exons represent ∼ 1% to 2% of the human genome,^58^ for the same amount of sequencing, WES can increase coverage or sample number. Cost effective and better suited to assess rare variants and diverse populations, low coverage sequencing (∼ 1×) combined to imputation represents a promising alternative to microarrays.^63^^,^^64^

**Long-read sequencing:** Bypassing the need for polymerase chain reaction, third-generation sequencing based on long reads reduces the risk of amplification-related errors and biases. By sequencing DNA fragments over 1 Mb long,^65^^,^^66^ this technology can accurately resolve complex regions, call haplotypes, and map structural variants (e.g., copy-number variants).^67^ Long-read sequencing can also detect base modifications,^68^ providing further insights into the analyzed sequence.

**CRISPR-based genome editing:** CRISPR technologies allow to assess the impact of precise genetic modifications, ranging from point mutations^69^^,^^70^ to whole-chromosome deletions,^71^^,^^72^ in physiological settings. CRISPR tools are available for most model organisms, cell lines, and patient-derived cells, with a growing number of functionalities extending beyond simple gene editing.^73^, ^74^, ^75^ It is possible to generate whole-body or tissue-specific gene knockins and knockouts, combine both approaches to create humanized models by excising the endogenous gene and replacing it by its human version, or perform epigenetic editing. Several alterations can be induced simultaneously, opening the door to the study of epistatic interactions.

For PGx to embrace the genomic revolution, ways to effectively organize, store, and share newly generated knowledge were required, leading to the creation of PGx databases (Table 1). The further the field advances, the more it will rely on the presence of centralized, well-structured, and inter-connected resources. Search terms *pharmacogenetics*/*pharmacogenomics* returned over 30,000 PubMed entries published between 2000 and 2020, testifying how the genomic revolution allowed the collection of data and knowledge at an unprecedented scale.

**Table 1:** List of five major pharmacogenetics knowledge databases and resources

Name	Aim	Reference and website
CPIC: The Clinical Pharmacogenetics Implementation Consortium	facilitate the clinical implementation of PGx by generating curated, evidence-based, and updated guidelines that provide prescription recommendations for gene-drug pairs	Relling and Klein^76^, Relling et al.^3^, https://cpicpgx.org/
DGIdb: The Drug Gene Interaction database	bridge the gap between drug discovery and PGx by integrating data from other databases, such as PharmGKB, Therapeutic Target Database,^77^ DrugBank,^78^ etc.	Griffith et al.^79^, Freshour et al.^80^, https://www.dgidb.org/
PGRN: The Pharmacogenomics Global Research Network	PGx hub with the aims of • providing a platform for the PGx community • promoting and advancing research in PGx • bringing awareness to the importance of PGx Coordinates PharmGKB, CPIC, and PharmVar	Relling et al.^81^, https://www.pgrn.org/
PharmGKB: The Pharmacogenetics Knowledge Base	first centralized gene-drug interaction database aiming at linking genomic data to molecular and cellular phenotypes, as well as to clinical information	Whirl-Carrillo et al.^82^, Whirl-Carrillo et al.^83^https://www.pharmgkb.org/
PharmVar: The Pharmacogenetic Variation Consortium	provide a centralized repository for all PGx variants and a standardized nomenclature for PGx alleles	Gaedigk et al.^84^, Gaedigk et al.^85^, https://www.pharmvar.org/

### Genome-wide association studies

Genome-wide association studies (GWASs) are observational studies wherein a large set of genetic variants spread across the genome, typically single nucleotide variants (SNVs) assessed with microarrays (Box 3), is scanned to identify associations with a phenotype. Hence, GWASs mitigate biases inherent to CGSs, enabling data-driven discovery of unsuspected PGx variants.

#### The first pharmacogenomics GWASs

One of the first PGx GWASs found that each additional copy of the *SLCO1B1* rs4149056 T>C allele increased the odds ratio for statin-induced myopathy by 4.5 (95% CI: 2.6–7.7; p = 2 × 10^−9^; MIM: 604843).^86^ If the gene was known to encode a hepatic statin transporter,^87^ it had not been linked to the ADR. Due to the rarity of the ADR, the GWAS was conducted within the larger SEARCH RCT, which followed ∼12,000 participants exposed to different doses of statins over the course of 6 years, providing access to matched controls. Case number was doubled by considering both individuals with definite and milder myopathy, highlighting the importance of adequate phenotype definition. These steps were crucial in gathering enough participants to have the statistical power to detect associations. The challenge is exacerbated in PGx studies, as only a fraction of individuals take the drug of interest and even a smaller fraction might develop a given ADR. Review of 23 PGx GWASs conducted between 2007 and 2010 showed that, on average, drug-response GWASs had 570 participants and ADR GWASs a case/control ratio of 70/120.^88^ Estimates from 2015 to 2020 show that these numbers are increasing (median: 1,220 participants) but remain ∼10 times lower than for non-PGx GWASs.^20^ An indirect consequence is that fewer GWASs have investigated drug responses.^20^^,^^89^ To overcome this hurdle, PGx GWASs can be performed within the context of RCTs or conducted by international consortia (e.g., PGRN-RIKEN),^90^ facilitating the recruitment of large cohorts.

**Figure 3:**
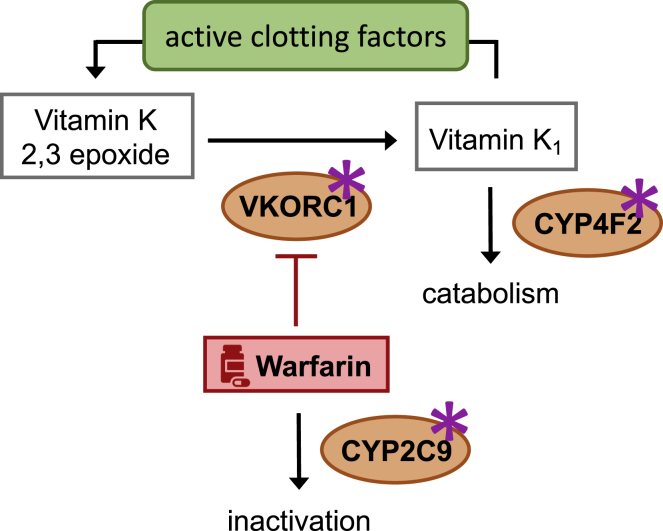
Warfarin pharmacogenetics Main enzymes (orange) encoded by genes whose polymorphisms (star) affect warfarin (red) dosage. Warfarin inhibits VKORC1, an enzyme that reduces vitamin K_1_ 2,3 epoxide to vitamin K, so that the latter can act as a cofactor for clotting factor activation. CYP2C9 is the main enzyme involved in the inactivation of warfarin. CYP4F2 is involved in vitamin K_1_ catabolism. Metabolites are in square boxes.

So far, most examples focused on ADRs, which are easy to identify but rare. Conversely, providing sufficient technical and financial means, drug response can be assessed in all treated subjects, offering greater statistical power to detect PGx interactions. One of the first drug responses investigated by GWAS was warfarin maintenance dose.^91^^,^^92^ At the time, warfarin was the most widely used oral anticoagulant, with prescription rates totaling 0.5%–1.5% of the population.^93^ The therapeutic window of warfarin is narrow and exhibits inter-individual variability, about 50% of which being explained by patient-specific demographics, clinical factors, and genetic background.^88^ Based on prior knowledge of warfarin metabolism (Figure 3), CGSs demonstrated the independent role of polymorphisms in *VKORC1* (MIM: 608547) and *CYP2C9* (MIM: 601130) in determining an individual’s drug dosage and ADR risk due to drug concentrations outside the therapeutic index.^94^ To determine whether other genes were implicated, a GWAS on warfarin dosage was conducted (n = 181), identifying a single genome-wide significant signal near *VKORC1*.^91^ A second GWAS with improved power (n = 1,053) identified significant signals for *VKORC1*, *CYP2C9*, and *CYP4F2* (MIM: 604426).^92^ The fact that these genome-wide studies did not reveal new genetic risk factors reflects that candidate genes with mechanistic support are bound to have strong PGx effect and will have the highest power to be detected by GWASs. Nevertheless, they corroborated earlier findings through an orthogonal method, excluding the presence of additional large-effect variants.

GWASs also discovered new PGx interactions. Before 2011, hepatitis C was treated with a poorly tolerated combination of pegylated interferons and ribavirin that failed to clear the infection in ∼50% of patients.^95^ Differential success rate across populations suggested a genetic origin for treatment efficacy (Box 2), prompting analysis through GWASs and revealing an association signal near *IFNL3* (previously *IL28B*; MIM: 607402, 609532).^25^^,^^96^, ^97^, ^98^ Arrival of direct-acting antivirals improved treatment success rate, especially in patients with unfavorable *IFNL3* genotype.^99^ Nowadays, new generations of well-tolerated and highly effective direct-acting antiviral regimens bypass the need for pegylated interferons altogether.^100^ This highlights an alternative to PGx screening: identifying drugs that elicit a more homogeneous response. Not always available, this alternative can lead to other issues, as new antivirals are associated with drug-drug interactions,^101^ themselves influenced by PGx. Together, GWASs are an important complement to hypothesis-driven CGSs and have discovered or replicated 586 unique drug-variant associations in the 14 years since the first published PGx GWASs.^20^

#### Polygenic pharmacogenomics

A long unanswered question relates to the genetic architecture of PGx traits. In 1918, R.A. Fisher showed that continuous traits can be explained through Mendelian inheritance of many genetic variants with a genuine, albeit small, effect.^102^ Since, the polygenic nature of complex traits has become widely accepted and pushed to the extreme with the omnigenic model, which hypothesizes that all genes expressed within a phenotype-relevant cell type contribute to the phenotype in question.^103^^,^^104^ Despite the quantitative nature of drug responses, early GWASs pointed to a mono- or oligogeneic architecture governed by few large-effect-size variants detectable by CGSs or small GWASs. Demonstrating polygenicity in PGx traits has not been straightforward. In the 2000s, CGSs implicated several genes in the response to the antidiabetic drug metformin,^105^, ^106^, ^107^, ^108^ but these loci were not sufficient to explain the estimated 34% heritability of the drug response.^109^ Instead, chromosome-wise heritability estimates pointed at the presence of multiple small-effect variants spread across the genome.^109^ In the following years, independent, better-powered GWASs implicated an increasing number of genes in metformin response,^110^, ^111^, ^112^, ^113^ supporting the predicted polygenic architecture. More recently, heritability of 12 PGx traits was estimated between 5% and 59%, with 62%–95% of the heritability being attributed to moderate- and small-effect variants.^114^ These results suggest a highly polygenic architecture for these traits, indicating that larger GWASs are likely to reveal further associations.

Improving genotype-based patient classification, polygenic scores (PGSs) have gained popularity to estimate the cumulative effect of genome-wide genetic variation on a phenotype. Given their success in identifying individuals at risk for common diseases,^115^ it is of interest to determine whether PGSs can help predict an individual’s drug response. So far, applications in PGx are sparse and have yielded mixed results, as highlighted in a 2021 comprehensive review of 51 PGx PGS studies.^116^ One of the most widely studied trait is response to antidepressants. Despite being an excellent candidate trait given the large number of treatment-refractory patients^117^ and a high heritability,^118^ only recently was its polygenic basis confirmed.^118^^,^^119^ Other studies started demonstrating the usefulness of PGx PGSs. Prior knowledge was used to design a PGS for ADR risk to the previously discussed anti-platelet drug clopidogrel.^52^ Six SNVs associating with on-treatment platelet reactivity were used to build a predictive PGS for MACEs, despite none of the variants associating with the trait, thereby demonstrating the predictive value of small cumulative effects. Another study developed a series of PGSs for drug-induced liver injury (DILI),^120^ a rare and genetically determined ADR induced by a wide range of drugs.^121^^,^^122^ In addition to validation in an independent dataset, PGSs were tested in liver organoids and primary hepatocytes, revealing shared predictivity across different drugs. Cell-based and transcriptomics assays further provided mechanistic insights. Free from the ethical and technical considerations associated with human studies, this “polygenicity-in-a-dish” approach allows to investigate the downstream impact of diverse genetic profiles in a tissue-specific and environmentally controlled way. Similar approaches with tailored follow-up assays might be applied to study other PGx traits.

Beyond classical challenges (sample sizes, phenotype definition, integration of rare variants, etc.), limitations intrinsic to microarray data will have to be overcome to fully understand the genetic architecture of PGx traits. It is difficult to design probes for the highly polymorphic, repetitive, or complex regions many PGx-relevant loci map to (e.g., *CYP*s and *HLA*). Though newer arrays include more PGx content, they do not yet provide perfect coverage of known PGx alleles.^60^ Furthermore, microarrays designed to assess common variants in Europeans provide suboptimal coverage in other populations (Box 2). This is mitigated by new arrays optimized for population-specific or multiethnic studies and ever larger and more diverse reference panels (e.g., TOPMed Imputation Reference panel)^123^ that improve imputation quality across diverse ancestries. Lastly, microarrays are not suited to accurately assess rare variants,^124^^,^^125^ despite evidence suggesting that these might account for some of the missing heritability in PGx traits.^126^

### Next-generation sequencing in pharmacogenomics

Unlike genotyping, sequencing assesses the exact order and identity of all nucleotides within a DNA segment (Box 3), allowing the discovery of unreported genetic variants. With decreasing sequencing price,^40^ it becomes possible to (1) increase sequencing coverage, leading to improved accuracy, and (2) sequence individuals from diverse populations, leading to the identification of new genetic variants.

#### The human pharmacogenomic landscape

Next-generation sequencing (NGS) has widely been used to gauge the mutational landscape of known PGx genes. If non-targeted approaches, such as whole-exome sequencing (WES) or whole-genome sequencing (WGS) (Box 3) allow the unbiased assessment of the mutational spectrum among all human genes, resources can be optimized by evaluating with increased coverage a set of genes likely to yield new insights. For instance, PGRNseq, a capture panel that sequences 84 PGx genes with ultra-deep 500× coverage,^127^ will assess PGx variation in the electronic Medical Records and Genomics (eMERGE)-PGx project (n ≈ 9,000).^128^^,^^129^ Another study (n = 14,002) analyzed rare variants in 202 drug target genes, suggesting that rare variants are both numerous and population specific.^130^ Genetic characterization of the 57 human *CYP* genes revealed over 6,000 new SNVs.^131^ Similar studies sampling individuals from various ethnicities were later conducted for a broader spectrum of PGx genes, coming to the conclusion that >90% of SNVs in these genes are rare.^132^^,^^133^ Despite their low frequency, rare variants strongly contribute to functional variability,^126^^,^^132^ many being predicted to be deleterious.^17^

At fixed effect size, sample size required to detect an association increases with decreasing allele frequency, so that GWASs lack the power to detect rare variant associations. This led to the development of statistical methods for cumulative rare variant association testing^134^ (Box 4), but few PGx studies have made use of them so far. Recently, optimal unified sequence kernel association test (SKAT-O) was used on WGS data to identify genetic determinants for bronchodilators response in 1,441 asthmatic children of diverse ethnicities (Box 2).^26^ Five significant SKAT-O regions were identified at non-ADME loci, explaining 4%–8% of the phenotypic variation. This exemplifies how new PGx discoveries can be made by promoting diversity and investigating rare variants in genes lacking prior known PGx interactions.

Box 4 **Rare variant association testing in pharmacogenomics**

**Burden tests** collapse rare variants within a predefined region or gene into a genetic score. As they falsely assume the same direction and magnitude of effect for all grouped variants, they can be inaccurate. For instance, variants in *CYP2C9* can increase, decrease, or abolish CYP2C9’s ability to metabolize warfarin.^135^

**Variance-component tests** investigate rare and common variants within a region simultaneously and have increased power in the presence of opposite effect variants or when only a small fraction of the variants is causal. This comes at the cost of requiring larger sample sizes, which can be challenging in PGx. The best known variance-component test is the sequence kernel association test (SKAT).^136^

**Combined tests** combine features from both above-mentioned tests, increasing robustness and performance in small sample sizes. The most widely used is the optimal unified SKAT test (SKAT-O).^137^

#### The non-coding pharmacogenome

Despite most GWAS signals mapping to non-coding regions, fine-mapping and mechanistic interpretation of these signals remains difficult.^138^ In the previously discussed association between *SLCO1B1* and statin-induced myopathy, the variant originally identified by the GWAS was intronic. Only after linkage disequilibrium (LD) analysis was the likely causal missense variant, rs4149056, identified.^86^ PGx associations mapping to non-coding regions could be explained by synthetic associations, a concept referring to the apparent association of a common marker with a trait resulting from that marker being in LD with one or multiple unobserved, rare, causal variants.^139^ Drug-response-associated non-coding variants could simply tag the deleterious mutational burden in corresponding PGx genes,^140^ even if it has been argued that synthetic associations created by rare variants are unlikely to explain most GWAS results.^141^

Non-coding variants can mechanistically influence drug response, e.g., by influencing the binding of drug-regulated transcription factors. This concept is demonstrated by a variant (rs4743771 C>A) affecting the response to rosiglitazone,^142^ an antidiabetic drug associated with adverse effects, such as increased cholesterol levels.^143^ Rosiglitazone mediates its therapeutic effect by binding and activating the nuclear receptor PPARγ (MIM: 601487),^144^ which induces the expression of multiple genes, including the cholesterol efflux transporter *ABCA1* (MIM: 600046).^145^ Chromatin immunoprecipitation sequencing showed that A/A carriers lack a rosiglitazone-induced PPARγ-binding site and fail to induce expression of nearby *ABCA1* upon administration of the drug.^142^ Confirming the clinical relevance of the variant, diabetic patients with the corresponding genotype experienced a reduced rosiglitazone-induced increase in cholesterol levels.^142^ Overall, combining NGS to biochemical and molecular assays can shed light on PGx variants in regulatory regions. Another emerging mechanism of action is via long non-coding RNAs (lncRNAs) modulation.^146^ While this area of research remains in its infancy, it was shown that the transcription factor HNF1α (MIM: 142410) and the lncRNA HNF1α-AS1 form a regulatory network that controls the expression of several *CYP* genes.^147^^,^^148^

#### Elucidating complex pharmacogenomic regions

Obtaining fine-scaled sequencing of PGx genes residing in complex genomic regions remains challenging. Previously mentioned *CYP2D6* is notoriously difficult to analyze with NGS due to the presence of homologous pseudogenes and a propensity for structural rearrangements.^149^ The gene is also highly polymorphic, with 149 alleles listed on PharmVar. Long-read sequencing workflows (Box 3) have been implemented to resolve *CYP2D6* haplotypes and diplotypes.^150^, ^151^, ^152^, ^153^, ^154^ Besides recovering known polymorphisms, these studies report a large fraction of new variants. As CYP2D6 metabolizes ∼25% of commonly prescribed drugs and that *CYP2D6* genotype directly influences drug metabolism efficiency,^149^ obtaining accurate diplotypes could improve *CYP2D6* metabolizer phenotype prediction. While long-read sequencing technologies offer exciting perspectives and first population-scale applications are emerging,^155^ they remain expensive and limited by low throughput and high error rates.

### Experimental pharmacogenomics

**Figure 4:**
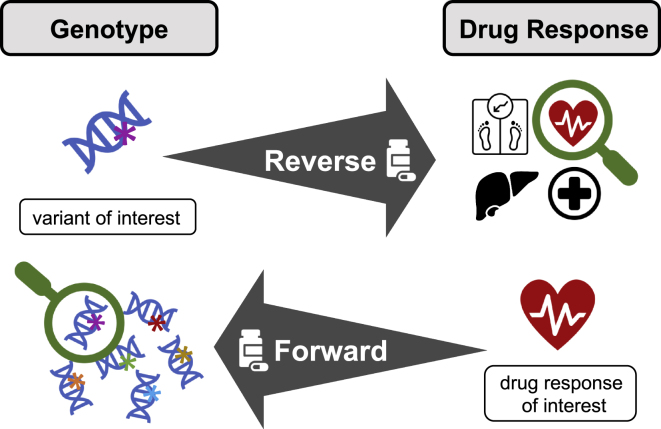
Schematic representation of two experimental approaches to pharmacogenetics The reverse genetics approach starts with a genetic variant of interest and aims at characterizing its phenotypic consequences upon drug exposure. Conversely, the forward genetics approach starts with a phenotype of interest and screens a genetically diverse population to identify genes or variants eliciting the studied response upon drug exposure.

Experimental settings provide precise control over genetic and environmental factors (e.g., diet, lifestyle, medication, and combinations thereof) and reduce the influence of confounders,^156^ making it possible to establish causal genotype-phenotype relationships and study complex phenomena, such as gene-by-gene (i.e., epistasis), gene-by-environment, and gene-by-sex interactions. The importance of understanding these effects is highlighted by an epidemiological study that found female sex and polypharmacy as the strongest predisposing factors for ADRs.^157^ Pharmacological differences between sexes have long been acknowledged, and there is emerging evidence that these might influence PGx.^158^^,^^159^ Moreover, polypharmacy might impact ADR risk through drug-drug interactions^160^ and drug-drug-gene interactions.^161^ Model organisms offer a convenient setting to disentangle these complex interactions through two experimental strategies (Figure 4).

#### A reverse genetics approach to pharmacogenetics

Reverse genetics investigates the phenotypic impact of a specific polymorphism by itself or in different sexes, genetic backgrounds, tissues, and/or environments. Before 2012, reverse genetics relied on laborious cloning techniques. Since, clustered regularly interspaced short palindromic repeats (CRISPR)-CRISPR-associated protein 9 (Cas9)^162^ revolutionized genetic engineering through its simplicity, versatility, and precision (Box 3) and over 50 publications already made use of CRISPR to induce full PGx gene knockouts or characterize specific variants.^163^ For instance, CRISPR-induced deletion of two linked SNVs in an upstream enhancer of *CYP2D6* revealed which one was responsible for increasing the expression of the gene.^164^^,^^165^ CRISPR technologies have been linked to off-target mutagenesis at sites with high sequence similarity^166^—which are particularly common among ADME genes^167^—even if careful usage of the tool should mitigate this issue.^168^ More importantly, the large number of PGx variants and persistent increase in drug prescription and polypharmacy^169^ render it impossible to assess the full combinatorial space of experimental settings at current experimental throughput, limiting usage of CRISPR to strong candidate loci.

#### A forward genetics approach to pharmacogenetics

Forward genetics exploits genetically diverse population maintained under controlled environmental conditions to facilitate the identification of genetic modifiers of drug response. Contrasting with targeted approach, CRISPR technologies can also be used to perform high-throughput gene-level genome-wide knockout, knockdown, or knockin screens to assess the low-dimension combinatorial effect of genetic perturbations. Screens can be coupled to high-throughput molecular techniques, such as bulk or single-cell RNA sequencing, allowing measurement of downstream consequences of induced mutations.^74^ Since drug resistance or susceptibility measured as cell proliferation or death can easily be assessed and exhibits limited tissue specificity, CRISPR screens have primarily been applied to cancer PGx research.^170^ In the future, screens could characterize a wider array of PGx-relevant readouts and move toward the investigation of specific SNVs. Deep mutational scanning takes a step in this direction by probing the function of thousands of variants in a single experiment^171^ and was recently applied to generate abundance and activity scores for 87% of the possible missense variants in *CYP2C9*.^172^ Providing unprecedented functional insights into the mutational landscape of the gene, these data can be used to train functional prediction algorithms (Functional prediction of rare variants). Another source of genetic diversity are genetic reference populations, which are available for most model organisms (e.g., mouse).^173^ Producing genetically identical individuals, they represent powerful mapping tools allowing the *in vivo* multi-tissue characterization of drug responses and can be coupled to extensive phenotyping and follow-up studies.^174^ An *in vitro* alternative that directly mimics human genetic diversity is to harvest patient cells, which can be studied as primary cells or induced into pluripotent stem cells to generate organoids, as in the previously described drug-induced liver injury PGS study.^120^ Patient-derived organoids can mimic an increasing number of organs and tissues, rendering the technology apt for personalized medicine strategies aiming at selecting the most efficient and safe treatment.^175^ While widespread clinical implementation awaits, it was shown that organoids from cystic fibrosis patients can predict drug efficacy.^176^^,^^177^

By studying appropriate and genetically diverse model systems, questions that were unanswerable due to ethical, logistical, or technical concerns become solvable. Experimental studies have their limitations, such as high costs, limited throughput, and/or poor reproducibility and translatability,^178^^,^^179^ but the virtuous cycle between human observational studies and *in vitro* and *in vivo* experimentation in model organisms^156^ should incite PGx to exploit the respective strengths of these approaches.

## Pharmacogenomics in the era of big data

### Electronic health records and biobanks

Electronic health records (EHRs) have become widespread over the past decade as, besides supporting and improving diagnosis, clinical decisions, and treatment coordination, they provide new data analytics opportunities.^180^ EHRs commonly encompass patient demographics, medical history, drug prescriptions, and in some cases laboratory results, radiological images, and wearable device data.^181^ Integration of EHRs is key to the realization of precision medicine, and current research already benefits from it by conducting nonrandomized studies.^182^, ^183^, ^184^, ^185^ Cost effectiveness, possibility to investigate rare diseases, large cohorts allowing stratification, and analysis of co-occurring conditions are some of the advantages over classical RCTs.^180^

#### Electronic health records in pharmacogenomics

Increasingly, biobanks match genotype entries with EHRs, broadening the phenotype query space exploitable for PGx research.^186^ Table 2 lists major research initiatives and biobanks for which EHRs are available. The UK Biobank (UKB) recently added primary care data for ∼230,000 participants, including consultation reports, medical diagnoses, and medication prescription.^205^ FinnGen plans to recruit ∼500,000 individuals by 2023 with access to EHRs from national health registries comprehending medication usage.^203^ Similarly, the Estonian Biobank contains detailed information about drug purchase and disease incidences collected since 2000.^183^^,^^187^ In parallel, many initiatives focus on the clinical implementation of PGx,^8^ such as the American eMERGE-PGx project,^128^ the European Ubiquitous Pharmacogenomics (U-PGx) consortium,^206^ the South East Asian Pharmacogenomics Research Network (SEAPharm),^207^^,^^208^ or the African Pharmacogenomics Consortium (APC).^209^ Because of the increased genetic diversity observed in African populations and their exposure to different types of diseases (e.g., higher infectious disease burden), the latter initiative is promising to unravel new PGx findings.

**Table 2:** List of 10 major biobanks and initiatives suitable for pharmacogenetic research

	Recruitment	Sample size	Sequencing data	EHR data	Omics data	References	IHCC	Notes
Estonian Genome Project	2002–	200,000	genome-wide genotyping (n = 200,000); WES (n = 2,500); WGS (n = 3,000)	n > 40,000	transcriptomics (n = 600; blood); metabolomics (n = 11,000); methylomics (n = 800); microbiomics (n = 2,500)	Leitsalu et al.^187^, Leitsalu et al.^188^, https://genomics.ut.ee	yes	in 2018–2019, increased from 50,000 to 200,000 samples
eMERGE	2007–	>135,000	genome-wide genotyping (n > 105,000); WES (n > 3,700); WGS (n > 1,700); PGRNseq (n = 9,000^a^)	all participants are linked to EHRs	None	Gottesman et al.^189^, Bush et al.^129^, https://emerge-network.org	yes	network of multiple distinct cohorts
UK Biobank	2006–2010	500,000	genome-wide genotyping (n = 500,000); WES (n = 500,000); WGS (n = 500,000^b^^,^^c^)	n = 230,000	blood biomarkers (n = 500,000); proteomics (n = 53,000^b^); metabolomics (n = 120,000); telomere length (n = 500,000)	Elliott and Peakman^190^, Bycroft et al.^191^, Backman et al.^192^, https://www.ukbiobank.ac.uk	yes	
DiscovEHR	2007–	>250,000	genome-wide genotyping (n > 150,000); WES (n > 100,000)	all participants are linked to EHRs	blood biomarkers	Carey et al.^193^, Dewey et al.^194^, http://www.discovehrshare.com	yes	part of Geisinger’s MyCode Community Health Initiative
Million Veteran Program	2011–	>1,000,000^b^	genome-wide genotyping (n = 1,000,000^b^^,^^d^); WES^b^; WGS (n = 100,000)	all participants are linked to Veteran Affairs EHR	blood biomarkers (n = 1,000,000^b^); proteomics^b^; methylomics^b^	Gaziano et al.^195^, Hunter-Zinck et al.^196^, https://www.mvp.va.gov	yes	
Taiwan Biobank	2012–	200,000^e^	genome-wide genotyping (n = 200,000^e^^,^^f^); WGS (n = 1,500)	national EHRs available^g^	blood biomarkers; metabolomics^e^	Wei et al.^197^, Lin et al.^198^, https://www.twbiobank.org.tw	yes	
H3Africa	2012–	>100,000	genome-wide genotyping^h^; WES^h^; WGS^h^ (n > 400)	None	blood biomarkers; microbiomics^h^	H3Africa Consortium et al.^199^, Mulder et al.^200^, https://h3africa.org	no	H3Africa is composed of several cohorts
Tohoku Medical Megabank Project	2013–2017	150,000	genome-wide genotyping (n = 150,000^b^); WGS (n = 14,000)	EHRs from MMWIN back-up system	transcriptomics (n = 100; blood); proteomics (n = 500); metabolomics (n = 46,000); methylomics (N = 100)	Tadaka et al.^201^, Ido et al.^202^, https://www.megabank.tohoku.ac.jp	no	composed of a population community-based cohort and a Birth and Three-Generation Cohort
FinnGen	2017–2023	500,000^i^	genome-wide genotyping (n = 500,000^i^^,^^j^)	all participants are linked to national EHRs	None	Locke et al.^203^, https://www.finngen.fi	no	
All of Us	2018–	>1,000,000^b^	genomic assays^b^	all participants are linked to EHRs	biological assays^b^	The All of Us Research Program Investigators^204^, https://www.researchallofus.org	yes	

The IHCC column informs whether the cohort is part of the International HundredK + Cohorts Consortium.

a eMERGE-PGx Project

b Planned.

c 150,000 samples released in 2021.

d 460,000 samples released in 2020.

e Planned in the population community-based cohort.

f 100,000 samples released in 2021.

g Not yet crosslinked.

h Sample sizes and available measurements are cohort dependent.

i By 2023.

j 350,000 samples released in 2022.

EHR-coupled biobanks accelerate our understanding of PGx by enabling the replication of known interactions and catalyzing new discoveries. Associations between drug maintenance dose and 9 PGx genes were tested in 200,000 UKB participants by assigning individuals to a metabolizer class (e.g., poor, intermediate, and normal) based on their genotype, revealing known *CYP2C9* and a novel *CYP2C19* variant as determinants for warfarin dosage.^184^ Another UKB study discovered an association between statin use and *NAT2* (MIM: 612182) genotype.^210^ The first study making use of WGS-coupled EHRs was conducted in ∼2,500 Estonian Biobank participants.^183^ Increasing sample size to ∼16,000 by including individuals with imputed genotypes, six known and nine new PGx interactions were identified.^183^ Importantly, many biobanks follow participants longitudinally, so that the number of individuals exposed to a drug or reporting an ADR increases with time. Initiatives such as the International HundredK+ Cohorts Consortium (IHCC) aim at creating a platform to harmonize data and facilitate information sharing across cohorts.^211^ Specifically, one of IHCC’s core initiatives is to “improve understanding of variability in response to treatments and identify novel PGx associations.” With ∼50 million participants from >100 cohorts,^211^ statistical power largely surpasses the one of traditional RCTs, making it likely for combined biobank studies to establish new PGx interactions.

#### Leveraging observational data to gain causal insights

**Figure 5:**
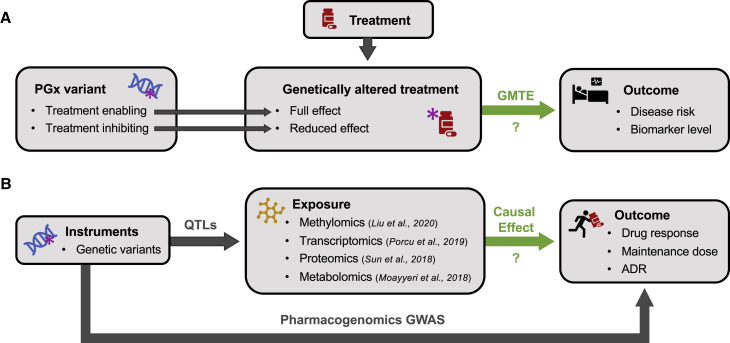
MR-analogous frameworks to infer causal effects in pharmacogenetic studies (A) Framework proposed by Bowden et al.^214^ to estimate a genetically modified treatment effect (GMTE) (green arrow), which describes the reduction in treatment effect experienced by patients with a PGx variant (treatment inhibiting) as opposed to those without (treatment enabling) the variant whilst on the same treatment. (B) Proposed framework to identify causal molecular mediators of PGx effects. Genetic variants are used as instrumental variables, intermediate phenotypes (e.g., methylomics, transcriptomics, proteomics, or metabolomics data) as exposure, and drug responses as outcome. The causal effect of the exposure on the outcome (green arrow) is estimated thanks to (1) the effect of the genetic variants on the exposure, measured by means of quantitative trait loci (QTLs), and (2) the effect of the same genetic variants on the outcome, measured by means of PGx GWAS.

Biobank studies follow the design of nonrandomized trials. Despite high agreement between treatment effects in randomized and nonrandomized trials,^212^ correlations between drug responses and genetic variants do not guarantee causality. Propensity score matching reduces bias from concomitant confounding variables through inclusion of selected covariates,^180^ and randomization between study participants is mimicked based on known observed covariates. Alternatively, Mendelian randomization (MR) approaches account for unobserved confounders,^213^ where the causal effect of a modifiable risk factor on an outcome is inferred from genetic associations coming from observational data (i.e., GWAS summary statistics or individual-level data). Analogously to RCTs, individuals are stratified based on the presence of genetic variants affecting the exposure of interest. MR was recently extended to study PGx through the triangulation within a study (TWIST) framework,^214^ which estimates a genetically moderated treatment effect (GMTE) and allows to calculate the reduction in treatment effect experienced by patients with a particular PGx variant, as compared with individuals lacking the variant (Figure 5A). Applying TWIST to the UKB revealed that *CYP2C19* LOF carriers experienced a 0.28% increased risk of stroke (p = 0.048) while on clopidogrel.^214^ As the TAILOR-PCI RCT failed at detecting a significant benefit to *CYP2C19*-based anti-platelet prescription (Randomized controlled trials), increased power resulting from larger sample size (clopidogrel n = 7,483; no clopidogrel n = 198,868) and consideration of a larger spectrum of LOF alleles could have contributed to the positive results. A different study analyzing statin usage in the UKB found that females homozygous for the *SLCO1B1∗5* allele known to impair intracellular statin uptake^215^ would have 0.147 mmol/L lower total cholesterol were they treated with a lipid-lowering medication unaffected by their genotype,^216^ reiterating the importance of sex as a modulator of PGx interactions. Overall, MR studies provide *in silico* orthogonal lines of evidence for the functional consequence of PGx variants and represent valuable tools to prioritize RCTs.

#### Challenges of large-scale biomedical databases

Increased usage of EHR-linked biobanks has brought attention to new challenges. First, EHRs frequently consist of unstructured, noisy, sparse, and incomplete clinical notes. As manual review by clinical experts is not scalable, automated solutions based on natural language-processing algorithms are required to extract informative features.^217^ Second, the massive size of biobanks necessitates sufficient storage capacities and computational resources to conduct analyses.^218^ This issue is exacerbated by the sensitive nature of the data: whereas patient privacy should be ensured, access to the data should be facilitated for the research community. With varying legislation across countries and differing views on acceptable privacy levels, development of data storage and management solutions is ongoing work.^219^ Finally, validating the increasing number of putative PGx interactions resulting from data-driven approaches is likely to become a major bottleneck. Conventional methods, such as RCTs, are inherently slow and low throughput, calling for alternative ways to validate putative PGx findings.

### Integration of multi-omics data

Biotechnological advances foster the generation of “omics” datasets, including methylomics, transcriptomics, proteomics, and metabolomics, which quantify DNA methylation, RNA expression, protein expression and modifications, and metabolite abundance, respectively.^220^ As disruptions in intermediate layers might be predictive of therapeutic outcomes (i.e., drug responses), exploring and integrating changes in biomarkers could help understand and predict pharmaco-omics interactions.

#### Molecular modeling of pharmaco-omics interactions

GWASs have shed light on the genetic architecture of complex traits but fall short on elucidating molecular mechanisms,^221^ as they identify associations between traits and blocks of co-inherited variants in LD, hindering fine-mapping. This challenge is addressed by methods integrating GWAS results with other omics data. Colocalization approaches prioritize putative causal genes based on shared signals between GWAS and omics data.^222^^,^^223^ Similarly, transcriptome-wide association studies (TWASs) pinpoint genes whose differential expression is associated with a given phenotype.^224^^,^^225^ Starting from genetic variants, gene expression is inferred from expression quantitative trait loci (eQTLs) and a derived gene expression PGS is correlated to the investigated trait. MR provides a framework that can be generalized to any modifiable exposure, including the transcriptome,^226^ proteome,^227^^,^^228^ methylome (CpG sites),^229^ or metabolome.^230^ Not widely employed in PGx,^231^ these methods improve our understanding of the molecular basis of association signals and could provide a corroborating source of evidence for novel PGx interactions (Figure 5B).

Recent studies highlighted the benefits of including biomarkers to machine-learning (ML) algorithms predicting drug response^232^ for antidepressant use,^233^ precision psychiatry,^234^ and warfarin dosage^27^ (Box 2). In the latter study, assuming that warfarin dosage depends on genetic variation altering both the expression and activity of key enzymes (Figure 3), tissue-specific eQTLs were used to infer the expression of 116 genes implicated in warfarin pharmacokinetics and pharmacokinetics and construct prediction models, which improved predictive performance by an additional 8%–12% of variance.

#### Limitations to multi-omics modeling in pharmacogenomics

Despite promising results, most predictive models are not ready for the clinics. Limited size and number of available multi-omics datasets increase the risk of model overfitting and prevents independent replication, respectively.^235^ Furthermore, study results are often siloed and only few databases with unbiased drug-screening experiments are publicly available.^236^ There is a need for large-scale *in vitro* and *in vivo* perturbation experiments measuring drug effects on multiple omics layers,^237^ as results from these assays could open new avenues to study biomarker combinations and drug-drug-gene interactions.^161^ Importantly, omics layers are not confined entities. Instead, each forms a small portion of an inter-connected biological system, so that testing every possible drug-biomarker combination in isolation would not provide a complete picture. System genetics strategies have been proposed to elucidate molecular mechanisms,^221^^,^^238^ but they remain exploratory and often lack the drug-omics interaction dimension. Toward this direction, a “multiscale interactome” was constructed, compiling public data on interactions between drugs, diseases, proteins, and biological functions to identify disease treatment mechanisms.^239^ System-level PGx is likely to become more elaborate in the future (Perspectives in mechanistic approaches), providing mechanistic explanations for pharmaco-omics interactions.

### *In silico* pharmacogenomics

#### Functional prediction of rare variants

The wealth of rare variants in known PGx genes discovered by NGS makes it challenging to infer their function through classical methods. Not only are *in silico* approaches necessary, but they can advance PGx faster and beyond what can be achieved with traditional methods. Functional impact of common variants can be characterized through association studies, *in vitro* heterologous expression systems, or computational prediction tools. However, these typically fall short when assessing rare variants.^240^ Computational algorithms usually rely on evolutionary conservation and use training sets with annotated pathogenic variants. Often under lower evolutionary constraints than disease-associated variants, PGx variants can be functional without being pathogenic.^240^ One solution is to engineer cell lines to harbor these variants and estimate their effect on enzymatic activity in a high-throughput and cost-effective manner, as was recently done for >8,000 *CYP2C9* variants^172^ following a forward genetics approach (A forward genetics approach to pharmacogenetics). Generated data can be used to train ML algorithms, yielding models applicable to clinical data. For instance, an ensemble ML model combining multiple functionality prediction algorithms was trained based on the enzymatic activity of 337 deleterious variants across 43 ADME genes, significantly increasing performance accuracy compared with single ML algorithms.^241^

Variant combinations—within the same or different genes—can also affect drug response. Employing deep learning algorithms, haplotype effects were investigated to predict CYP2D6 enzymatic activity.^242^^,^^243^ Training a convolutional neural network based on star alleles definitions, *in vitro* enzymatic activity of both known and unknown *CYP2D6* haplotypes could be predicted.^242^ Similarly, a neural network was trained on a prospective cohort of breast cancer patients to predict CYP2D6-mediated tamoxifen metabolism.^243^ In this second study, omitting star allele haplotype assignment yielded better prediction scores, implying it might be time to move beyond star allele PGx. Both studies being limited in their scope, investigating a broader set of genes is warranted to draw general conclusions.

#### Perspectives in mechanistic approaches

We previously introduced statistical methods that integrate GWASs’ findings with other datasets to identify causal biomarker-drug response relationships. Assuming a biomarker alteration is causal to a disease, identifying drugs achieving their therapeutic effect by inducing the opposite change should provide viable treatment options. This hypothesis was tested by searching for drugs inducing gene expression patterns opposite to the ones observed in seven psychiatric disorders within the Connectivity Map (CMap),^244^ a library containing the expression profile of 1,000 genes upon perturbation by ∼20,000 small molecules.^245^ Although there is no protein analogue to CMap, ChEMBL, the largest database of curated drug targets,^246^ and DrugBank^78^ form valuable resources to conduct similar studies at the proteome level. Drug repositioning is a topic on its own,^247^ but this example showcases an application of a mechanistic understanding of drug-disease interactions. In the context of PGx, this strategy could identify drugs counteracting patient-specific, disease-causing biomarker changes.

**Figure 6:**
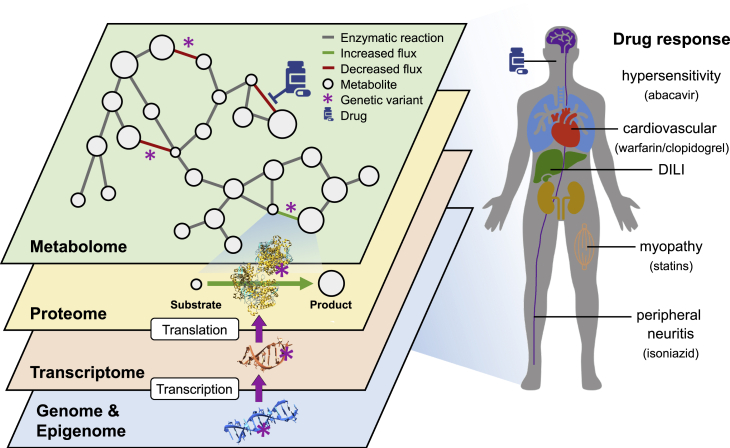
A mechanistic systems biology view on pharmacogenomics The metabolic network of each cell is composed of metabolites (nodes) connected by enzymatic reactions (edges). It is modulated by genetic and epigenetic variants (star) that propagate through several intermediate molecular layers (e.g., transcriptome and proteome), as well as by drug intake. This network and its modulation are cell type specific and influence an individual’s drug response and ADR risk, as illustrated on the right with examples of differential drug responses manifesting themselves in specific tissues.

Alternatively to data-driven top-down strategies are bottom-up approaches, such as the ones used in systems biology.^248^ Systems biology aims at constructing metabolic network models describing complex biological systems,^249^ with nodes representing metabolite concentrations connected by mathematical equations defined by enzyme kinetics. Since enzyme kinetics is at the core of drug metabolism,^250^ it would be relevant to understand how consequences of genetic variants propagate through the biochemical network (Figure 6). Network effects can be calculated through flux balance analyses, using mathematical models accounting for thermodynamic constraints and mRNA and protein abundances.^251^ Efforts to reconstruct a genome-scale network are concerted in Recon3D, the most comprehensive human metabolic network to date.^252^ Recon3D provides information on 13,543 enzymatic reactions and 4,140 metabolites and describes the relationships between involved genes, proteins, the reactions these proteins catalyze, and links between genetic variants and metabolic effects. Integration of genetic variation into metabolic studies remains in its infancy, but the ever-growing supply of diverse datasets is leading to a steady increase in our understanding of disease mechanisms. There is a great demand for refined multiscale modeling, as expressed in a review authored by the pharmaceutical industry, with the hope that more realistic *in silico* models will improve prediction of drug mechanisms and safety before human clinical testing.^253^

## The past and future of pharmacogenetics

Seventy years of methodological development have transformed PGx from an emerging science into an interdisciplinary area of research key to the implementation of personalized medicine. Pharmacogenetics established the role of genetics in pharmacological responses, with RCTs validating the first CGSs discoveries. Completion of the Human Genome Project triggered the transition to pharmacogenomics: sequencing technologies allowed ascertainment of the human PGx landscape while genome-editing tools enabled discovery and functional validation of PGx interactions. Finally, tools and concepts from statistics and computer science have been and will be key to analyze and integrate ever more complex datasets, bringing us a step closer to the *in silico* study of pharmaco-omics.

Despite indisputable progress, numerous challenges lay ahead. Throughout the review, we highlighted areas of research we imagine will be decisive for the future of PGx and that overlap challenges recently laid out.^254^ Due to its interdisciplinary nature, progress in PGx can be achieved through different avenues, including data generation, data analysis and integration, and discovery prioritization and validation. Size and diversity of information contained in biobanks is constantly increasing, as development of sequencing and omics technologies improves genetic and molecular pathway understanding, while EHRs broaden clinical characterization. In parallel, high-throughput screens in genetically engineered cell lines, patient-derived organoids, and model organisms can assess the impact of genetic variations in controlled but tunable environmental settings. Establishment of efficient and secure solutions for data storage and sharing will foster the development of statistical and ML approaches aiming at analyzing and integrating data originating from various sources, resulting in a better understanding of the genetic architecture of PGx traits and mechanistic insights. RCTs will likely continue to play an important role in the clinical implementation of PGx, but it will be important to maximize their success rate and improve their throughput. Creative *in vitro* and *in vivo* experiments, as well as high-quality *in silico* mechanistic and predictive models, can generate robust hypotheses, thereby improving prioritization of new putative PGx interactions. Awareness and incorporation of this knowledge in RCT design might increase their success rate and/or promote development of companion diagnostics gauging the suitability of a treatment for a given patient.^255^

Solutions to the above-mentioned challenges should ensure that progress in PGx benefits all. Genomic research remains predominantly conducted in populations of European ancestry, despite general agreement that more diversity is needed to understand the human genome.^24^^,^^256^, ^257^, ^258^, ^259^ This particularly applies to PGx, as many PGx genes are under weak global selective pressures and exhibit population-specific allele frequencies.^15^, ^16^, ^17^, ^18^, ^19^^,^^130^^,^^260^ In this optic, *All of US* will recruit one million US citizens to build the largest and most diverse cohort in history, marking an important shift from the paradigm of studying white-male individuals to representing the full spectrum of a globalized world, including its minorities.^204^ Another core value of the project is to strengthen ties between participants and researchers to return value to all parties and ensure knowledge flow from bench to bedside and back. Acknowledging that most health care systems currently lack the infrastructure to implement screening for even well-established PGx variant, we argue that genetically-guided prescription, together with the recording of health outcomes through EHRs, will fuel PGx research while helping refine prescription guidelines. This virtuous cycle will benefit from increased diversity in both research and clinical settings, as the richness in genetic variation will accelerate mechanistic understandings of PGx, whose findings can in turn be generalized to other populations.

## Web resources

ClinicalTrials.gov, https://clinicaltrials.gov/.
CPIC, https://cpicpgx.org/.
IHCC, https://ihccglobal.org/.
OMIM, https://www.omim.org/.
PharmVar, https://www.pharmvar.org/.
PubMed.gov, https://pubmed.ncbi.nlm.nih.gov/.
